# The genomic diversity of arthropod-specific viruses reinforces the continental distribution pattern of *Aedes aegypti*

**DOI:** 10.1186/s13071-025-07120-3

**Published:** 2025-11-18

**Authors:** Weimar D. Briñez, Daniel Alfonso Urrea, Marina Muñoz, Luz H. Patiño, Juan David Ramírez

**Affiliations:** 1 Centro de Investigaciones en Microbiología y Biotecnología-UR (CIMBIUR), School of Sciences and Engineering, Universidad del Rosario, Bogotá, Colombia; 2 Laboratorio de Investigaciones en Parasitología Tropical (LIPT), Facultad de Ciencias, Universidad del Tolima, Ibagué, Colombia; 3 Instituto de Biotecnología-UN (IBUN), Universidad Nacional de Colombia, Bogotá, Colombia; 4 Center for Global Health and Interdisciplinary Research, Department of Global, Environmental and Genomic Health Sciences, College of Public Health, University of South Florida, Tampa, FL USA

**Keywords:** Phylogeography, Insect vector, Recombination, Arboviruses, Biological evolution

## Abstract

**Background:**

*Aedes aegypti* is the primary vector of arboviruses worldwide, including dengue, Zika, chikungunya, and yellow fever. It is believed to have originated in Africa and migrated to the Americas during the sixteenth and seventeenth centuries, subsequently spreading to Asia and Oceania between the nineteenth and twentieth centuries. These mosquitoes harbor insect-specific viruses (ISVs), which represent the majority of their core virome. Phasivirus phasiense (PCLV), cell-fusing agent virus (CFAV), and *Aedes* anphevirus (AeAV) stand out for their global distribution in vectors. Within this framework, this study aims to evaluate whether ISVs can provide insights into the continental dispersal history of *Ae. aegypti*.

**Methods:**

A total of 96 complete sequences from three ISVs (CFAV, AeAV, and PCLV) were analyzed. These were obtained from wild *Ae. aegypti* collected across four regions: the Americas (45), Asia (38), Africa (12), and Oceania (1), as well as from laboratory colonies originally derived from wild mosquitoes. Of the 45 sequences from the Americas, 12 were newly assembled for this study from mosquito samples collected in Ibagué, Colombia in 2021 (4 per ISV). To reconstruct the vector’s dispersal history and compare evolutionary patterns between viruses with and without evidence of recombination, multiple methodological approaches were used: (1) phylogenetic analyses with BEAST1 to estimate divergence times, (2) statistical tests for recombination (*Φ*-test), (3) recombination network construction using SplitsTree and RDP, and (4) principal component analysis (PCA) to evaluate population structure.

**Results:**

The analysis of three ISVs in global *Ae. aegypti* populations allowed for the inference of the vector’s historical dispersal. The results revealed: (1) genetically structured diversity patterns associated with geography, (2) evidence of recombination in PCLV, but not in AeAV and CFAV, and (3) contrasting temporal estimates suggesting multiple introductions into the Americas between the seventeenth and nineteenth centuries, as well as recent dispersal into Oceania.

**Conclusions:**

ISVs are promising tools for studying the dispersal and evolution of *Ae. aegypti*, although their viral dynamics can influence their effectiveness as evolutionary markers. CFAV, with recombination evidence, reflects strong connectivity among populations. AeAV, despite lacking recombination but high variability, provides accurate insights into recent dispersal. PCLV, with low diversity and regional recombination, is useful for analyzing local population structures.

**Supplementary Information:**

The online version contains supplementary material available at 10.1186/s13071-025-07120-3.

## Background

It is estimated that *Aedes aegypti* originated during a period of drought in North Africa around 4000–6000 years ago, in what is now the Sahara Desert [1, 2]. This desertification process likely facilitated the “domestication” of *Ae. aegypti*, as human water storage practices provided artificial breeding sites, while the proximity to these human populations offered a stable and accessible blood source [2–4]. Approximately 500 years ago, *Ae. aegypti* likely arrived in the New World through the transatlantic slave trade and later expanded into Asia and the Pacific Islands, including Australia [5].

To support this hypothesis, several methodologies have been used to reconstruct the phylogeographic history of *Ae. aegypti*, including genomic analyses, single-nucleotide polymorphism (SNP), and microsatellite studies [5–8], as well as mitochondrial DNA analyses to infer patterns of divergence and dispersal [6, 9–11]. These approaches are generally useful for determining the evolution of population structure at regional scales and over long periods [12]. However, new techniques are being implemented to analyze these evolutionary processes on a local scale and over shorter time frames. These approaches are primarily aimed at understanding the biology, ecology, and population dynamics of *Ae. aegypti*, which in turn informs our understanding of the emergence and transmission of the arboviruses it vectors, such as dengue (DENV), Zika (ZIKV), chikungunya (CHIKV), and yellow fever (YFV) [13]. In this context, outbreak dynamics are studied by integrating spatial, temporal, and viral genetic data, along with information on human and vector mobility. Following this approach, it may be possible to use circulating viruses to infer the phylogeography and distribution of *Ae. aegypti* [12].

In this regard, insect-specific viruses (ISVs) are exclusive to insects and do not require a vertebrate host to complete their life cycle [14, 15], meaning that their presence in *Ae. aegypti* is generally stable and not influenced by external epidemiological factors [12]. ISVs have maintained long-term associations with their vectors, with interactions ranging from neutral coexistence to possible effects on mosquito biology and arbovirus dynamics [16]. Their persistence in *Ae. aegypti* populations has been reported worldwide [15], and they experience lower selective pressure compared with arboviruses [16], which may result in more stable evolutionary rates and a lower probability of mutational saturation [17].

Overall, ISVs represent approximately 73.8% of the mosquito virome [18]. Among them, Phasi Charoen-like virus (PCLV), from the family Phenuiviridae, constitutes between 60% and 98% of the *Ae. aegypti* core virome globally [18–20]. Likewise, cell-fusing agent virus (CFAV), the first ISV identified in 1970, belongs to the Flaviviridae family, along with DENV, ZIKV, and YFV. Its 11 kb genome encodes a single polyprotein, and its abundance in the core virome of *Ae. aegypti* ranges between 2% and 11% [17–19, 21]. Another relevant ISV is *Aedes* anphevirus (AeAV), from the Xinmoviridae family, which has an 11 kb genome and a core virome abundance ranging from 0.04% to 0.14% [22–24]. PCLV, CFAV, and AeAV are widely distributed across all continents, suggesting a stable and long-standing relationship with their host [15, 18, 19, 21, 22].

Given that ISVs have coevolved with *Ae. aegypti*, their analysis can represent a promising tool for reconstructing the evolutionary history and dispersal routes of the vector, complementing traditional genetic studies [22, 25, 26]. In this sense, although ISVs have been widely detected in *Ae. aegypti*, their intraspecific genetic variability, recombination processes, and evolutionary relationships among them, given the worldwide dispersal of the vector, have not been explored on a regional scale, nor has their potential as markers of *Ae. aegypti* dispersal. To fill this gap, our study evaluates ISVs from a genomic approach, analyzing their genetic variability across five continents, assessing whether ISVs can reflect the phylogeographic patterns of *Ae. aegypti*, offering a novel perspective that complements traditional genetic methods. To this end, we analyzed a total of 96 complete genomes from three ISV species—PCLV (L segment), AeAV, and CFAV—comprising 12 novel sequences generated for this study from wild *Ae. aegypti* collected in Ibagué, Colombia, and 84 publicly available sequences retrieved from the National Center for Biotechnology Information (NCBI).

## Methods

### Sample collection and RNA sequencing

During 2021, *Ae. aegypti* populations were collected in urban areas of the municipality of Ibagué, Tolima, Colombia (coordinates: 4°26′16″ N, 75°12′02″ W). Four pools of 50 females each were obtained from different districts within the municipality. To identify viral diversity in these populations, RNA extraction was performed for each pool using mechanical maceration with a mortar and liquid nitrogen as the homogenization method. This technique was selected owing to its advantages in preserving RNA integrity, avoiding fragmentation compared with other homogenization methods [27, 28].

For RNA extraction, the TRI Reagent^®^ protocol (Thermo Fisher Scientific) was used [28], adding 1.5 mL to each pool to maximize RNA yield and quality. Sample quality was assessed to ensure suitability for sequencing using the 2100 Bioanalyzer; the electropherogram was examined to record band sizes associated with the 18S- and 28S-rRNA peaks as a measure of RNA integrity. Library preparation was performed using the TruSeq Stranded Total RNA LT Sample Prep Kit (Gold) with rRNA depletion (Ribo-Zero H/M/R Gold). Sequencing was carried out on the Illumina NovaSeq 6000 platform, generating 100-base pair (bp) paired-end reads, with an average of 5.9 Gb of data for each of the four pools of 50 females processed, for a total of approximately 26 Gb of information.

### Bioinformatic processing

The four sequencing products were processed separately following the bioinformatic approach described below: Quality control was performed using FastQC version 0.11.9 [29]. Adapter sequences, low-quality bases (< Q30), and short reads (< 70 bp) were removed using Prinseq-lite version 0.20.4 [30] and Trimmomatic version 0.39 [31].

Subsequently, to analyze the virome, we first filtered out the mosquito transcriptome to reduce the number of reads to be analyzed. For this, each of the cleaned sequencing products was aligned using Hisat2 [32] against the *Ae. aegypti* reference genome available from NCBI and VectorBase [33] (Chromosomes [Chr] 1–3 and Mitochondrial: NC_035108.1, NC_035107.1, NC_035109.1, NC_035159.1). Reads that did not align to the vector genome were stored in a separate FASTQ file for each pool and individually assembled de novo using Trinity version 2.13 [34] with the following parameters: $Trinity-seqType fq-max_memory 240G-left 1.fastq.gz-right 2.fastq.gz-CPU 32.

Assembly metrics were analyzed using descriptive statistics with the script count_fasta_cnsg.pl [35]. This process aimed to obtain contigs that represented partial or complete viral genomes present in the samples.

The assembled contigs were compared via Basic Local Alignment Search Tool-nucleotide (BLASTn) against the nucleotide (nt) and nr (non redundant) NCBI databases locally [36], applying a stringent threshold with an *E* ≤ 1 × 10^−6^ and a maximum of 20 hits per query. This produced a list of candidate viruses present in each pool.

On the basis of the viruses identified in this list, reference genomes were downloaded from the NCBI database, selecting for each virus the corresponding official reference genome. Alignments for each virus per pool were performed using Hisat2 to confirm viral presence, coverage, and depth. Concordant reads for each virus per pool were de novo assembled using Trinity, applying the same parameters and criteria mentioned above. Once the list of contigs and their taxonomic assignments was generated, alignments, organization, and orientation of the contigs into a genome or pseudomolecule were performed using the Abacas script version 1.3.1 [37] on the basis of the consulted reference genomes.

These organized viral genomes were annotated using the automatic annotation transfer tool Rapid Annotation Transfer Tools (RATT) [38], using annotated reference genomes in EMBL format, and finally verified manually. Genome coverage and depth were evaluated using Samtools version 1.9 [39] and Qualimap [40]. The reads were subsequently deposited in the NCBI database under project ID PRJNA1257366.

### Identification of genomic sequences of *Aedes* anphevirus, Phasivirus phasiense, and cell-fusing agent virus

Given the complete genome recovery of PCLV, CFAV, and AeAV from each of the pools obtained through sequencing and bioinformatic processing of the samples collected in Ibagué, a dataset was constructed by supplementing with sequences available in the NCBI database (https://www.ncbi.nlm.nih.gov/) for each of these viruses.

For sequence selection, the reference genomes of PCLV (ASM281483v1) [41], CFAV (NC_001564.2) [42], and AeAV (NC_075274.1) [22] were used. A BLASTn analysis was conducted using the MegaBLAST algorithm, with an *E*-value threshold of ≤ 1 × 10^−6^ and a maximum of 5000 matches. All other parameters were kept at their default settings.

From these results, only samples with a minimum coverage of 91% relative to the reference genome were selected [43, 44] (Supplementary Table S1, “Coverage %” column). This threshold was chosen to ensure adequate representation of the viral genome while retaining a sufficient number of sequences for comparative analyses. This practice is standard in viral genomics, where strict 100% coverage cutoffs are rarely applied owing to the inherent variability of viral quasispecies and technical limitations that can create stochastic gaps in coverage [45]. Given the high mutation rates and frequent sequence variability in RNA viruses, a strict 100% coverage cutoff would have excluded many informative sequences, especially those derived from wild-caught samples. The 91% threshold thus provides a balance between data completeness and sample inclusion, a strategy consistent with methodologies used in large-scale viral surveillance and metagenomics studies [46], minimizing biases due to missing data while preserving geographic and viral lineage diversity. Subsequently, we included sequences obtained from wild *Ae. aegypti* collected across different continents and, when available, sequences from laboratory colonies originally established from wild populations of the same regions. The distribution of sequences by region and ISV was as follows: Africa (PCLV, 7; CFAV, 2; AeAV, 3), Americas (PCLV, 20; CFAV, 12; AeAV, 13), Asia (PCLV, 26; CFAV, 7; AeAV, 5), and Oceania (AeAV, 1). For PCLV, only the L segment was selected owing to its higher conservation and availability of sequences (Supplementary Table S1).

To ensure data quality, all reads were verified to originate from Illumina platforms or platforms with comparable Phred quality [47], minimizing sequencing errors. In the case of previously published sequences, the associated bioinformatic methodology was reviewed to rule out potential errors in data processing (Supplementary Table S1, “Technology” and “Assembly” columns). Once the database was compiled, each sequence was downloaded and classified by viral species, host, continent, country, locality, NCBI ID, percentage of coverage, sequencing technology, year of collection, genome size, and assembly technology (Supplementary Table S1).

### Alignment and phylogenomic analysis

To validate the data quality, their taxonomic identity, and their geographical relationships and associations, a phylogenetic analysis was carried out as follows: An alignment was performed using MAFFT version 7 software [48] with the FFT-NS-2 algorithm and default parameters. Subsequently, UTR regions and low-coverage areas were manually removed using UGENE version 33.0 [49], to ensure a proper comparison at the sequence level. These alignments were then saved in FASTA format.

On the basis of the alignment of each virus, a phylogenetic analysis was conducted using the maximum likelihood (ML) method on the IQ-TREE version 2 web server [50], employing the nucleotide substitution model automatically selected by ModelFinder [51]. The robustness of the topology was assessed with 10,000 bootstrap replicates [13]. A midpoint rooting was subsequently performed, and nodes with bootstrap values ≥ 70 were highlighted [21, 50, 51]. Finally, the geographic origin of the samples at the continental scale was color-coded using the iTOL version 6 web server [52].

### Genetic variability analysis

Subsequently, on the basis of the same alignments used for the phylogenomic analysis, viral genetic diversity was assessed using DnaSP version 6 software by applying the DNA polymorphism algorithm. The completeness of the region was verified, and nucleotide diversity (*π*) was calculated. This index measures the average number of nucleotide differences per site between pairs of sequences in the dataset, providing an estimate of genetic variation. In addition, owing to the high mutation rate characteristic of RNA viruses, the Jukes and Cantor correction was applied to account for the possibility of multiple substitutions at the same site, which may otherwise lead to underestimation of variability [53].

Watterson’s Theta (*Θ*_w_) and neutrality tests, including Tajima’s *D*, were also calculated to evaluate patterns of molecular evolution and to test for deviations from neutrality, such as population expansion or selection. The resulting data were organized into a table for further comparison. Standard deviations of Watterson’s Theta (*Θ*_w_), both per site and per sequence (*σ*^2^ < sub > *θ* < /sub >), were then computed using the corresponding formula:

$$SD\, = \,\sqrt {\theta_{W} }$$

where:

SD = standard deviation

*Θ*_w_ = central estimated value of divergence

Subsequently, a dissimilarity matrix (genetic distance) was generated using RStudio on the basis of the Jukes–Cantor algorithm, from the aligned sequences for each virus. This matrix was then used in RStudio to perform a principal component analysis (PCA) on the basis of the genetic distance matrix between sequences to identify clustering patterns. For the PCA analysis, the R libraries ape, adegenet, and ggplot2 were used.

### Population structure inference and recombination detection

A phylogenetic network was constructed for each alignment using default parameters in SplitsTree version 4 [54] to visualize complex evolutionary relationships and potential recombination events. In addition, the phi test, as implemented in SplitsTree version 4, was applied to confirm signals of recombination. Furthermore, a recombination analysis was performed using RDP version 5 [55], implementing multiple algorithms, including RDP, BootScan, MaxChi, Chimaera, SiScan, and 3Seq. Only recombination events with a significance threshold of *P* < 0.05 in at least three methods were considered [56].

### Bayesian inference and estimation of divergence times

To estimate the divergence times of PCLV, AeAV, and CFAV, as well as potential associations between their geographic distribution and the evolutionary history of the vector, a Bayesian phylogenetic inference was conducted using BEAST1 version 2.6.7 [57]. A relaxed log-normal molecular clock model was employed to allow for variable evolutionary rates across lineages. Temporal calibration of the tree was based on the collection dates of the sequences, spanning from 2002 to 2021, enabling estimation of the time to the most recent common ancestor (TMRCA). Model convergence was assessed through effective sample size (ESS) values greater than 200, ensuring robust parameter estimates.

A Bayesian skyline coalescent tree prior was used to flexibly reconstruct changes in viral population sizes over time. The nucleotide substitution model GTR + Γ4 + I was selected via ModelFinder [51], and an evolutionary rate of 1 × 10^−3^ substitutions/site/year, based on previous studies of similar RNA viruses, was applied [58]. All other parameters were inferred from the dataset. To evaluate spatiotemporal dispersion patterns, sequences were grouped by continent (America, Asia, Africa, and Oceania), allowing for the inference of global dispersal events.

Markov chain Monte Carlo (MCMC) chains were run for 100 million generations, with sampling every 10,000 iterations. Parameter convergence was assessed using Tracer version 1.7.1 [59], ensuring effective sample sizes (ESS) greater than 200. A 10% burn-in was applied, along with the options -limit 0.95 and -hpd2D 0.95, before summarizing the maximum clade credibility tree using TreeAnnotator version 2.6.7. Divergence times and the temporal structure of viral lineages were visualized using FigTree version 1.4.4, with each geographic origin color-coded. In addition, the time axis was set in reverse chronological order (negative direction).

### Estimation of standard deviation (SD) from the 95% highest posterior density (HPD) interval

To estimate the standard deviation (SD) of the age of a node on the basis of the 95% highest posterior density (HPD) interval, the following approximation was used, assuming a normal distribution. Since approximately 95% of the values in a normal distribution lie within ±2 standard deviations (2*σ*) of the mean, the width of the HPD interval can be approximated as four times the standard deviation.

Therefore, the standard deviation is estimated using the following formula:

$$SD = \frac{HDPsub - HDPinf}{4}$$

where:

SD = standard deviation

HPD_sup = upper limit of the 95% highest posterior density (HPD) interval

HPD_inf = lower limit of the 95% HPD interval

## Results

### Dataset

Sequences were retrieved from the NCBI database through BLASTn searches using the complete genomes of each studied virus as reference. Applying strict quality criteria, sequences derived primarily from wild *Ae. aegypti* samples with genome coverage ≥ 91% were selected; in some cases, sequences from laboratory colonies originating from wild populations were also included [43, 44]. In the specific case of PCLV, the analysis focused on the L segment owing to its higher availability of complete sequences.

**Fig. 1:**
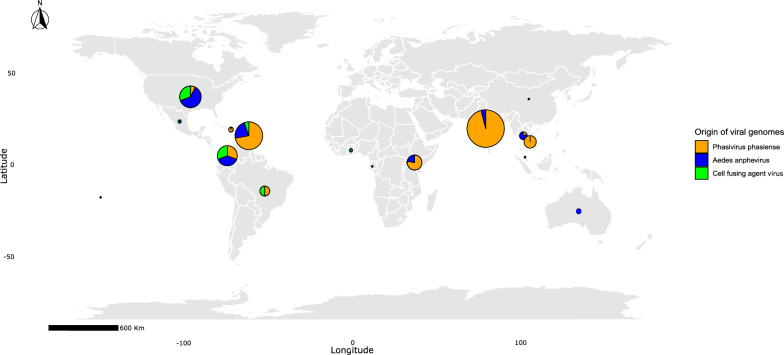
Map showing the geographical distribution of insect-specific viruses (ISVs), color-coded as follows: orange for Phasivirus phasiense (PCLV), blue for *Aedes* anphevirus (AeAV), and green for cell-fusing agent virus (CFAV). The sequences were obtained from vectors reported worldwide, with a total of 45 sequences from the Americas, 38 from Asia, 12 from Africa, and 1 from Australia. The size of each pie chart reflects the number of sequences obtained from each geographic region

Four complete sequences were generated in this study for each of the three viruses—PCLV, AeAV, and CFAV—from *Ae. aegypti* individuals collected in Ibagué, Colombia in 2021. These sequences were taxonomically assigned using BLASTn against the NCBI nt database and showed high genome coverage: 99% for PCLV, 96% for AeAV, and 98% for CFAV (Fig. 1 and Supplementary Table S1). In addition, publicly available sequences were retrieved for comparative purposes, resulting in a total dataset of 53 sequences for PCLV, 22 for AeAV, and 21 for CFAV. This filtering ensured that subsequent analyses were based on complete and comparable genomic data.

### Phylogenomic analysis

To validate the taxonomy, data quality, and evolutionary relationships among the evaluated viruses, an initial phylogenetic analysis was performed using the sequences obtained in this study alongside the reference genomes for the three viruses: AeAV (NC_075274.1), PCLV (NC_038262.1), and CFAV (NC_001564.2).

**Fig. 2:**
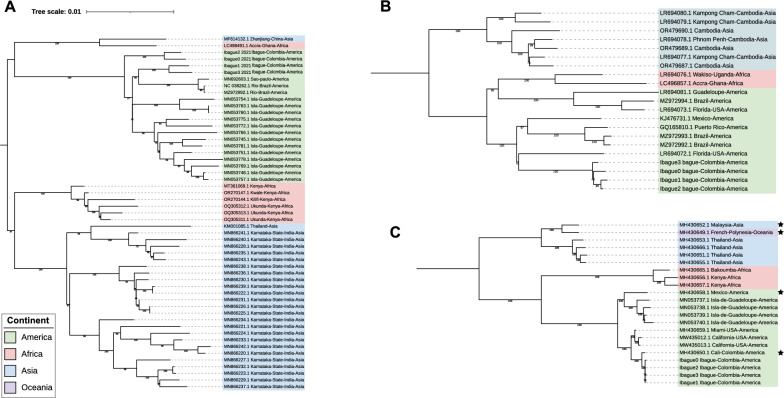
Maximum likelihood phylogenetic trees of *Aedes aegypti* insect-specific viruses (ISVs) inferred using 1000 bootstrap replicates (values > 70% shown at nodes). **A** represents Phasivirus phasiense (PCLV), **B** shows cell-fusing agent virus (CFAV), and **C** displays *Aedes* anphevirus (AeAV). Sequence identifiers correspond to GenBank accession numbers, with tips color-coded by geographic origin: green (America), blue (Asia), red (Africa), and yellow (Oceania). Samples derived from laboratory colonies are marked with a black star (⋆)

Overall, three distinct subgroups were identified for each virus on the basis of their geographic origin: Americas, Asia, and Africa (Fig. 2), revealing geographically structured dispersal patterns, whereas the AeAV sequence from Oceania exhibited closer affinity with Asian lineages (Fig. 2C). This suggests a recent introduction of these viruses (and consequently their mosquito vectors) into Oceania, likely originating from Asia. The analysis incorporated sequences from established mosquito colonies (Supplementary Table S1).

Another key observation is that the phylogenetic trees suggest that PCLV, CFAV, and AeAV populations in Africa and the Americas diverged from their Asian counterparts at an early point in their evolutionary history. However, as shown in Fig. 2, certain PCLV sequences from Asia and Africa appear to share a more recent common ancestor compared with those from the Americas and Africa. In particular, the Asian sequence MF614132.1 and the African sequence LC496491.1 suggest possible introductions of PCLV-infected vectors from Africa into Asia.

In addition, differences in the length of terminal branches were observed for all three viruses, even at local scales (i.e., sequences from the same geographic location). For example, in Fig. 2A, B, variation is evident among sequences collected in Ibagué in 2021.

### Genetic variability of AeAV, CFAV, and PCLV

To better understand the genetic variability of the studied viruses and their potential relationship with patterns of geographic dispersal, a genetic diversity analysis was conducted. This included the estimation of parameters such as nucleotide diversity, number of haplotypes, and the number of polymorphic sites, which allowed for the evaluation of the degree of differentiation among viral populations (Table 1). Intraspecific genetic variability for each virus (with *n* > 1 per continent) is detailed in Supplementary Table S2.

**Table 1:** Genetic diversity of evaluated ISVs

Parameter	*Aedes* anphevirus (AeAV)	Cell-fusing agent virus (CFAV)	Phasivirus phasiense (PCLV)
Number of sequences	22	21	53
Genome length (bp)	12207	4948	5796
Polymorphic sites	1871	864	912
Nucleotide diversity (*π* ± SD)	0.0470 ± 0.005	0.04217 ± 0.001	0.0258 ± 0.0006
Haplotypes (diversity)	21 (0.996)	21 (1.0)	50 (0.998)
*Θ*_w_ (per site ± SD)	0.04205 ± 0.013	0.04853 ± 0.016	0.03501 ± 0.009
*Θ*_w_ (per seq ± SD)	513.25 ± 165.84	240.111 ± 84.7	200.968 ± 56.2
Gaps/missing data	92	3	56

The table summarizes various genetic and diversity parameters for three viral species: Aedes anphevirus (AeAV), cell-fusing agent virus (CFAV), and Phasivirus phasiense (PCLV). The standard deviation to *Θ*_w_ per site and sequence is standard deviation of theta (no recombination)

AeAV exhibited high genetic diversity, with a nucleotide diversity (*π*) of 0.0470 and a Watterson’s Theta per site (*Θ*_w_) of 0.04205—closely aligned values that suggest a scenario of neutral evolution. A total of 1871 polymorphic sites were identified within a 12,207 bp region, reflecting substantial genetic variability. Haplotype diversity (Hd) was 0.996, with 21 haplotypes across 22 sequences, indicating that nearly all sequences were unique. The standard deviation (SD) of *π* was low (0.005), supporting the robustness of this estimate. The presence of 92 sites with gaps or missing data is not unexpected, given the nature of RNA viruses (Table 1).

Similarly, CFAV showed the highest genetic diversity among the viruses analyzed, with a *Θ*_w_ per site of 0.04853—the highest in the study. Nucleotide diversity (*π* = 0.04217) was comparable to that of AeAV, but haplotype diversity reached the maximum value (Hd = 1.0), as all 21 analyzed sequences corresponded to distinct haplotypes. Only three sites contained gaps, indicating a high-quality alignment. The low *π* SD (0.005) and the relatively high SD in *Θ*_w_ (0.016) suggest that while overall diversity is high, there may be subpopulations with distinct evolutionary dynamics (Table 1).

In this context, PCLV exhibited moderate diversity (*π* = 0.0258), lower than AeAV and CFAV, but with a high haplotype diversity (Hd = 0.998), as 50 out of 53 sequences represented unique haplotypes. The *Θ*_w_ per site (0.03501) was higher than *π*, which could indicate purifying selection or a recent population expansion. A total of 912 polymorphic sites were found in a 5796-bp region, along with 56 sites containing gaps. Although the low *π* SD (0.004) suggests consistent results, the presence of missing data indicates that subsequent analyses should be interpreted with caution (Table 1).

Intraspecific genetic variability for each virus (with *n* > 1 per continent) is detailed in Supplementary Table S2. To explore these patterns in greater detail, we performed region-specific analyses for the Americas, Asia, and Africa (Supplementary Table S2). These disaggregated results confirmed that AeAV exhibited its highest diversity in Asia (*π* = 0.0443), while CFAV was most diverse in the Americas (*π* = 0.03469). Haplotype diversity remained high across all viruses and regions, suggesting substantial intraregional variation.

### Geographic pattern of genetic variability

**Fig. 3:**
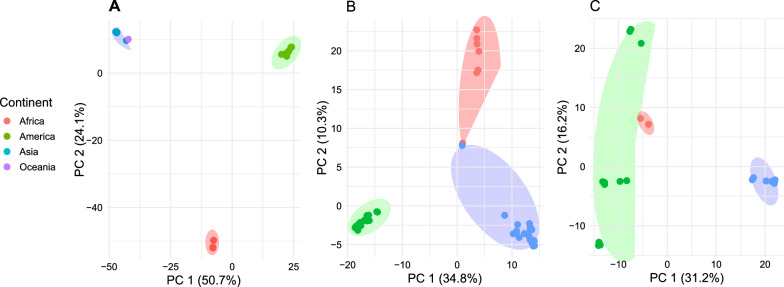
Principal component analysis (PCA) of *Aedes aegypti* insect-specific viruses: **A** *Aedes *anphevirus (AeAV), **B** Phasivirus phasiense (PCLV), and **C** cell-fusing agent virus (CFAV). Individual viral genomes are represented by points, and continental groups are indicated by elipses. Colors indicate geographic origin—green (America), blue (Asia), red (Africa), yellow (Oceania)—with points and ellipses sharing the same color but differing in opacity to distinguish individuals from group clusters. Distinct clustering by continent is observed, particularly among American, African, and Asian populations

High values of nucleotide and haplotype diversity reflect divergent evolutionary dynamics among the analyzed ISVs. To assess whether this genetic variability is structured by geographic origin, a principal component analysis (PCA) was performed, which revealed distinct viral groupings by continent (Fig. 3A–C). Neotropical populations showed marked genetic differentiation compared with Asian populations, while African populations exhibited partial overlap with other geographic groups (Fig. 3B, C). This pattern was particularly evident for PCLV, which displayed overlap between African and Asian samples. American populations exhibited greater genetic variability, with CFAV sequences showing variable positions along the second principal component (*y*-axis), including overlap with a single African sequence (Fig. 3C). In line with the PCA, the phylogenomic analysis also identified clear geographic structuring among the viral lineages (Fig. 2A), and the PCA results show a clear separation of continental clusters, consistent with limited genetic exchange between these regions.

### Exploration of other evolutionary events

**Fig. 4:**
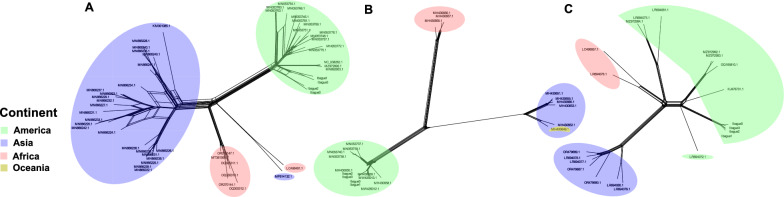
Phylogenetic networks of *Aedes aegypti* insect-specific viruses: **A** Phasivirus phasiense (PCLV), **B** *Aedes* anphevirus (AeAV), and **C** cell-fusing agent virus (CFAV). Sequences are color-coded by geographic origin: green (America), blue (Asia), yellow (Oceania), and red (Africa). The networks reveal: (1) distinct continental clustering (particularly America/Africa/Asia), suggesting phylogeographic structure; and (2) multiple reticulations in PCLV (**A**) and CFAV (**C**) networks, indicating genetic recombination events

The phylogenetic analyses conducted previously revealed the presence of three distinct lineages for each of the viruses studied. However, to avoid relying solely on strictly bifurcating methods such as phylogenetic trees and to explore potential recombination events, a phylogenetic network analysis was performed using SplitsTree version 4.19.2 (Fig. 4).

The network analysis (SplitsTree, Fig. 4) revealed the clustering of sequences into three divergent groups corresponding to their geographic origin, consistent with the results of the phylogenomic and PCA analyses. In both PCLV and CFAV, multiple phylogenetic reticulations were observed in the network, indicating ongoing genetic recombination processes. This was statistically supported for PCLV by the phi test (*P* = 2.116 × 10^−6^), while for CFAV the phi test result (*P* = 0.09765) did not reach significance, indicating only a weak or nonsignificant signal of recombination.

In contrast, AeAV exhibited a network with few reticulations (*P* = 0.9641), suggesting an absence of recombination. Specific relationships were identified between African and Asian PCLV sequences LC498491.1 from Ghana and MF614132.1 from China.

The RDP analysis detected recombination events in PCLV, which were mainly found among sequences from the same geographic origin, particularly in regions of the L segment (2821–5418 bp) and the RdRp gene (1572–3792 bp) (Supplementary Fig. S1).

### Estimation of TMRCA and temporal analysis of ISV spread in *Aedes aegypti*

On the basis of previous findings, we analyzed the temporal spread and time to the most recent common ancestor (TMRCA) of the ISVs CFAV and AeAV using TreeTime under molecular clock models. PCLV was excluded from this analysis, as recombination events in this virus occurred exclusively among sequences from the same geographic region, which may not reflect large-scale dispersal patterns. A similar number of available sequences for AeAV and CFAV enabled a balanced comparison between models.

Time-scaled analysis using TimeTree:

To estimate divergence times and explore the evolutionary history of ISVs in *Ae. aegypti*, time-calibrated phylogenetic trees were constructed using BEAST1.

**Fig. 5:**
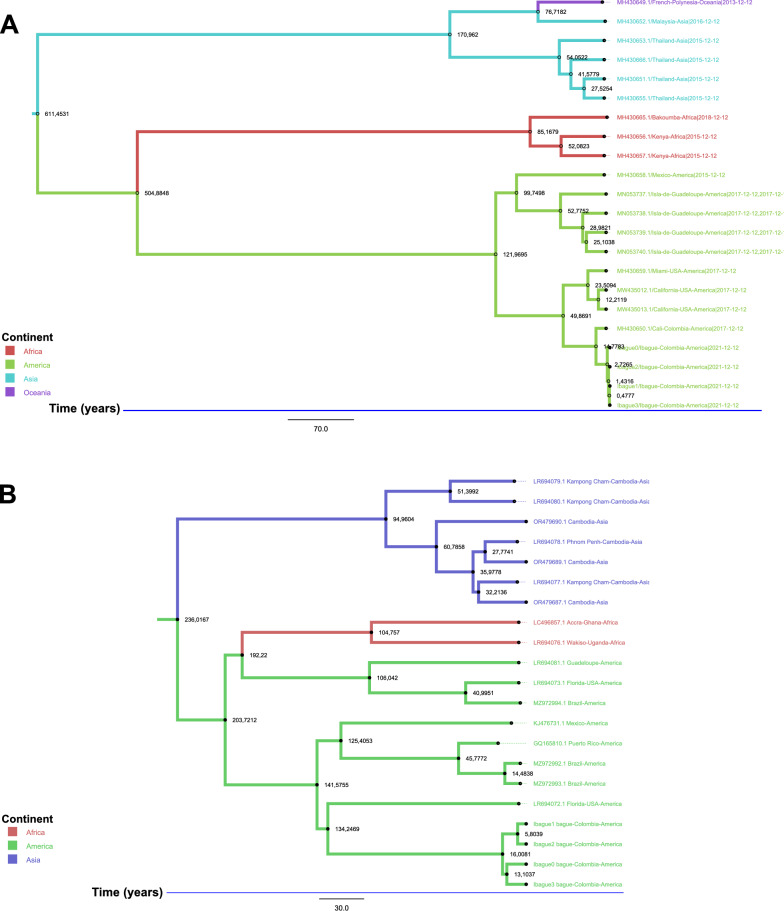
Time-calibrated trees (TimeTree) for *Aedes anphevirus* (AeAV) (**A**) and cell-fusing agent virus (CFAV) (**B**), generated using BEAST1. A relaxed molecular clock was applied for sequence analysis, while a strict clock was used for time scaling and geographic assignment. Model convergence was validated by ensuring an effective sample size (ESS) greater than 200, guaranteeing the stability of the inferred parameters. The phylogenetic topology was reconstructed using maximum likelihood methods, providing a reliable representation of evolutionary relationships. In addition, 1 × 10^9^ MCMC (Markov chain Monte Carlo) iterations were implemented to obtain precise estimates of divergence times and the evolutionary dynamics of both viruses

The time-calibrated phylogeny of AeAV revealed a clear geographic structure with well-defined separation between African, American, and Asian lineages (Fig. 5A). The analysis indicated an initial divergence event between Asian lineages and the African–American clade approximately 611 years ago (±194.9), followed by the separation of African and American lineages around 504 years ago (±90.8). Within the Asian lineage, sequences from Oceania diverged from a Malaysian lineage approximately 76 (±14.6) years ago, with Malaysian sequences showing the closest genetic affinity to Oceania strains.

The TimeTree analysis of CFAV revealed a distinct evolutionary pattern (Fig. 5B). Asian sequences diverged from the African and American lineages approximately 236 years ago (± 60.9). Subsequently, around 203 years ago, most American sequences and all African sequences diverged. Finally, approximately 192 years ago (± 109.7), a clear divergence event occurred where the American sequences (including those from Guadeloupe, Florida USA, and Brazil) completely separated from the African lineage, establishing distinct American and African clades.

## Discussion

Multiple studies have characterized the *Ae. aegypti* virome across diverse geographic regions, consistently identifying a core set of insect-specific viruses (ISVs), notably CFAV, AeAV, and PCLV [60, 61]. In parallel, recent research has explored the factors shaping the global distribution of *Ae. aegypti*, emphasizing the epidemiological relevance of understanding both natural and anthropogenic drivers of its dispersal [2, 62–64]. Herein, we integrated virome and empiric vector dispersal, providing evidence that ISVs associated with *Ae. aegypti* exhibit genetic diversity and empirical geographic structure that provides insights into the mosquito’s dispersal dynamics at a continental scale [12].

Our comprehensive analysis of three insect-specific viruses (ISVs)—CFAV, PCLV, and AeAV—revealed divergent evolutionary patterns. Recombination events were detected in PCLV but not in CFAV and AeAV. All three viruses exhibited high haplotype diversity, highlighting their evolutionary dynamism and potential as molecular markers of *Ae. aegypti* dispersal. As ISVs are exclusive to insect hosts and not influenced by vertebrate immune pressures or zoonotic cycles, they exhibit greater evolutionary stability and lower clearance rates [12, 16, 17, 65, 66]. Their long-term coevolution with *Ae. aegypti* positions them as potential proxies for investigating historical patterns of mosquito spread [67, 68]. Future efforts deepen the use of ISVs as phylogeographic tools, potentially incorporating additional viral taxa to strengthen inferences of vector movement across time and space.

Among the three viruses, CFAV demonstrated the highest nucleotide diversity (*π* = 0.0421) and haplotype diversity (Hd = 1.0; Table 1). Despite the presence of reticulations in the phylogenetic network, the phi test result (*P* = 0.09765) did not reach significance, indicating no strong evidence of recombination. These results build on previous work by Baidaliuk et al. [69] but differ in that the present dataset included sequences obtained directly from wild mosquitoes as well as from long-established laboratory colonies. While this approach avoids biases from extensive in vitro cell passages, we acknowledge that long-term laboratory maintenance can introduce its own artifacts, such as genetic drift or adaptation, and therefore, interpreted data from these colonies with caution. Phylogenetic and temporal analyses indicated the divergence of Asian lineages from the African–American cluster approximately 236 years ago (around the year 1785), followed by a separation between African and American lineages around 192 years ago (~1829; Fig. 5B). These estimates correspond to the late colonial and early post-colonial periods, when human-mediated trade and migration between continents intensified [2, 68]. CFAV thus holds promise as a molecular chronometer to trace historical *Ae. aegypti* movements. Expanding datasets with ancient samples or museum specimens could further refine these reconstructions.

AeAV showed no evidence of recombination (phi test *P* = 0.9641), yet exhibited the highest nucleotide diversity (*π* = 0.0470) and near-maximal haplotype diversity (Hd = 0.996; Table 1). Phylogenetic analysis revealed clear geographic structuring, with distinct African, Asian, and American clades (Fig. 2C), consistent with lineage diversification following geographic isolation. Temporal phylogenetic estimates revealed a deep divergence between African and Asian lineages approximately 611 years ago (~1410), and a subsequent split between African and American lineages ~504 years ago (~1517; Fig. 5A). These timings coincide with the early phases of European colonial expansion and the onset of the transatlantic slave trade [2, 70], processes historically implicated in the spread of *Ae. aegypti* across continents [5, 71]. The alignment between AeAV divergence and historical human-driven dispersal supports the potential of ISVs as robust phylogeographic markers for reconstructing long-term mosquito movements.

Notably, the divergence times estimated from our ISV phylogenies present a different chronological order compared with the established dispersal history of *Ae. aegypti*, which is based on the vector’s own genomic markers. While mosquito genomics indicates a dispersal from Africa to the Americas followed by introductions into Asia [5, 8, 72], our viral data suggest an earlier divergence of the Asian lineages. We do not interpret this discrepancy as a contradiction but rather as evidence that ISVs provide a complementary layer of information. It is plausible that the evolutionary history of these viruses is not only shaped by the deep, historical migrations of their host but also by more recent or transient contact events between mosquito populations that did not result in significant host gene flow [73]. Therefore, ISV phylogeography may not simply recapitulate the vector’s history but rather enriches it, offering insights into ecological and epidemiological links that are not apparent from host genetics alone.

A key advantage of using ISVs as phylogeographic markers is that their evolutionary dynamics are distinct from those of their mosquito host. With potentially faster mutation rates and different selection pressures [12, 16, 17, 65, 66], ISVs can act as high-resolution trackers for recent or transient events, such as inter-population contacts that may not result in lasting host gene flow. This is precisely how they complement the information from the mosquito genome: While the host’s nuclear DNA reveals deep, long-term migration and isolation history [5, 8, 72], ISVs provide a more dynamic ledger of ecological and epidemiological connections. Our findings, which show a different historical narrative for the viruses compared with the vector, underscore this complementary role.

PCLV presented the lowest nucleotide diversity (*π* = 0.0256) among the three viruses, although haplotype diversity remained high (Hd = 0.998; Table 1). Recombination was detected within geographically restricted lineages (Supplementary Fig. S1), especially in the L segment (positions 2821–5418 and 1572–3792), consistent with modular evolution observed in segmented viruses [74]. The phi test confirmed significant recombination (*P* = 4.162 × 10^−5^). These patterns support the hypothesis that PCLV may be more suitable for studying fine-scale dispersal dynamics rather than broader transcontinental movements [75]. However, the exclusion of the M and S genome segments—often more variable—may underestimate total viral diversity [76]. Full genome sequencing, especially of the underexplored M and S segments, could reveal cryptic diversity in PCLV and improve its utility in vector tracking at multiple spatial scales.

Differences in the evolutionary trajectories of these ISVs may be attributed to intrinsic viral traits such as replication rate, interactions with the mosquito immune system, and vertical transmission modes [77, 78]. In addition, the degree of virus–vector coadaptation likely influences their suitability as molecular markers. Viruses such as AeAV, with deeper divergence times, may better reflect ancient mosquito dispersal, while CFAV and PCLV appear more informative of recent or regional movements (Hollingsworth et al. [12]). Dissecting the molecular interactions between ISVs and *Ae. aegypti* will provide deeper insight into their coevolution and potential as stable evolutionary indicators.

Our findings indicate that ISVs, owing to their potentially faster evolutionary rates, may capture recent and fine-scale population events that are often invisible to host-based genomic markers. For instance, the recombination detected within geographically restricted PCLV lineages is indicative of ongoing, localized viral evolution. Similarly, the more recent divergence estimates for CFAV, corresponding to the post-colonial era, suggest its potential utility in tracking mosquito dynamics on shorter time frames. Collectively, this suggests that ISVs may function not only as proxies for ancient history but also as potential high-resolution tools for understanding contemporary vector dispersal.

Our phylogenetic findings resonate with prior studies: Parry and Asgari [22] identified three monophyletic AeAV lineages corresponding to Africa, the Asia-Pacific region, and the Americas, consistent with our results and further supported by the single Oceanian sequence, which clustered within the Asian clade. Zhou et al. [79] observed shared ancestry between African and American CFAV strains, in line with our observations. Lole et al. [75] noted PCLV phylogeographic associations such as Ghana–China and USA–UK, also aligning with our results. These similarities bolster the robustness of our analyses and emphasize the reproducibility of ISV phylogeography across studies. Cross-validation of ISV phylogenies with mosquito population genomics could further strengthen inferences of vector movement and evolutionary history.

The timing of CFAV’s diversification in the New World, with divergence events estimated at ~1785 and ~1829 (236 and 192 years ago, respectively; Fig. 5B), coincides with the late colonial and early post-colonial periods, when intensified trade and human mobility linked Africa and the Americas [2]. This reinforces its value as a marker for recent mosquito dispersal. In contrast, AeAV’s elevated variability and deeper divergence times (~1410 and ~1517; Fig. 5A) suggest that it may better capture historical mosquito movements associated with early transoceanic exchanges. Although PCLV’s segmented genome poses interpretative challenges, its localized recombination signatures offer unique insights into microevolutionary processes. Integrating viral and vector genomic data with historical and ecological records can enhance the accuracy of dispersal modeling in vector-borne disease systems.

A major strength of this study lies in the reliance on sequences obtained directly from wild-caught mosquitoes, complemented by data from long-established laboratory colonies originally derived from field populations, which minimizes potential laboratory-induced biases [79]. By employing a comparative approach across ISVs with varying genome architectures and evolutionary rates, we demonstrate the potential of combining phylogenomics, recombination analysis, and molecular-clock dating to track vector dynamics. To our knowledge, this is among the first studies to holistically assess multiple ISVs as indirect indicators of global mosquito mobility. This approach may serve as a blueprint for future vector studies seeking to integrate molecular epidemiology with evolutionary ecology.

Nonetheless, some limitations must be acknowledged. Geographic sampling was uneven (Americas: 45 sequences; Asia: 38; Africa: 13; Oceania: 3; Fig. 1), which may skew regional inferences and limit the resolution of phylogeographic patterns. The dataset is particularly biased by the underrepresentation of Africa and Oceania—two regions that were central to the historical dispersal of *Ae. aegypti*. This limitation must be taken into account when interpreting phylogenetic splits involving these regions, as it could underestimate their contribution to global virus diversity. Partial genome representation (e.g., only the L segment of PCLV) may also mask additional diversity or recombination signals [76]. While PCLV exhibited clear evidence of recombination, CFAV showed only weak signals and AeAV none, underscoring the need for broader genomic coverage and comparative analyses. The absence of prior studies on recombination in AeAV and PCLV further restricts contextual interpretation. Recombination may act as a compensatory mechanism in RNA viruses to mitigate deleterious mutations—a hypothesis supported by the “Müller’s ratchet” model [80]. Future work should prioritize balanced sampling from underrepresented regions, along with full-genome analyses and functional assays of recombination, to better resolve the evolutionary forces shaping ISV diversity.

Finally, these findings carry important implications for vector surveillance and arbovirus risk prediction. ISVs may reflect colonization history and mobility of *Ae. aegypti*, offering early warning proxies for arbovirus outbreaks. Principal component analysis (PCA) revealed clear continental clustering (Fig. 3), particularly in the Americas and Asia, while African sequences were more diffuse—potentially due to complex migration histories or gene flow. By mapping ISV phylogeography, we can refine vector control strategies, especially in regions undergoing rapid urbanization or environmental change. Future studies should aim to integrate these molecular insights with real-time vector surveillance to predict and preempt emerging public health threats.

A promising perspective lies in the isolation and experimental propagation of ISVs, which would enable controlled studies to directly estimate mutation rates, recombination frequencies, and adaptive dynamics—parameters currently inferred indirectly from metagenomic datasets [81]. While metagenomic studies offer broad insights into viral diversity and distribution, they often rely on consensus sequences and pooled samples, potentially underestimating intrahost variability and transient viral subpopulations. In contrast, isolating ISVs and passaging them under defined conditions (e.g., in cell lines or live mosquitoes) would allow precise measurement of evolutionary rates, identification of mutational hotspots, and testing of selective pressures in real time [82]. This approach could clarify whether observed phylogenetic divergence reflects true mutation accumulation or artifacts of sequencing and assembly pipelines [83]. Moreover, comparing mutation rates derived from isolation studies with those inferred from metagenomics would help validate molecular clock models and refine the use of ISVs as tracers of mosquito population history [12].

## Conclusions

Our findings highlight the utility of insect-specific viruses (ISVs) as informative molecular markers that complement and enrich our understanding of the evolutionary and dispersal history of *Ae. aegypti*. Our multi-virus approach reveals a multilayered historical narrative: AeAV provides a window into ancient dispersal events linked to early colonial exchanges, while CFAV tracks more recent dynamics from the post-colonial era, and PCLV illuminates localized, microevolutionary processes. This demonstrates that it is the integration of these complementary viral histories that allows for a more holistic reconstruction of vector movement across different spatial and temporal scales. Beyond methodological innovation, this virome-based framework offers a novel lens for monitoring mosquito mobility and anticipating future patterns of vector spread in an era of accelerating climate change and global connectivity. Ultimately, harnessing the information encoded in the virome represents a powerful, next-generation strategy for the surveillance and control of one of the world’s most important disease vectors.

## Supplementary Information

Additional file 1. Fig. S1. Recombination analysis of *Phasivirus phasiense* (PCLV).

Additional file 2. Table S1. Geographic distribution and genetic data of insect-specific viruses (ISVs) in *Ae. aegypti*.

Additional file 3. Table S2. Genetic diversity parameters of ISVs by continent.

## Data Availability

Sequence data that support the findings of this study have been deposited in the NCBI Sequence Read Archive (SRA) under BioProject accession no. PRJNA1257366.
