# Supplementary material for: The genomic diversity of arthropod-specific viruses reinforces the continental distribution pattern of Aedes aegypti
Source: Parasit Vectors. 2025 Nov 18;18:468. doi: 10.1186/s13071-025-07120-3 (PMC12625191; doi:10.1186/s13071-025-07120-3)
Supplement: Supplementary file 1 — Additional file 1. Fig. S1. Recombination analysis of Phasivirus phasiense (PCLV). [file 13071_2025_7120_MOESM1_ESM.pdf]

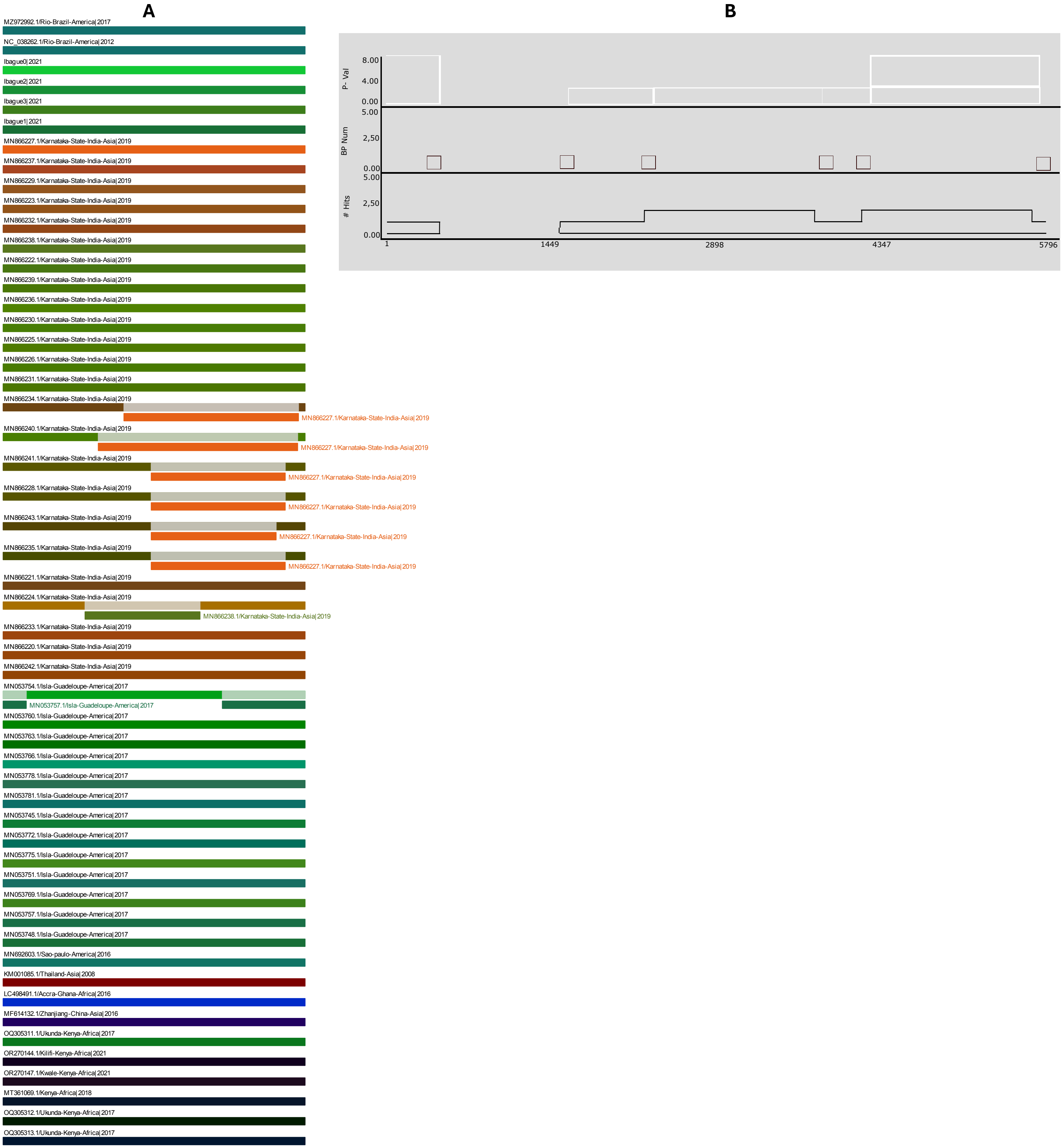
A
B
MZ972992.1/Rio-Brazil-America|2017
NC_038262.1/Rio-Brazil-America|2012
Ibague0|2021
Ibague2|2021
Ibague3|2021
Ibague1|2021
MN866227.1/Karnataka-State-India-Asia|2019
MN866237.1/Karnataka-State-India-Asia|2019
MN866229.1/Karnataka-State-India-Asia|2019
MN866223.1/Karnataka-State-India-Asia|2019
MN866232.1/Karnataka-State-India-Asia|2019
MN866238.1/Karnataka-State-India-Asia|2019
MN866222.1/Karnataka-State-India-Asia|2019
MN866239.1/Karnataka-State-India-Asia|2019
MN866236.1/Karnataka-State-India-Asia|2019
MN866230.1/Karnataka-State-India-Asia|2019
MN866225.1/Karnataka-State-India-Asia|2019
MN866226.1/Karnataka-State-India-Asia|2019
MN866231.1/Karnataka-State-India-Asia|2019
MN866234.1/Karnataka-State-India-Asia|2019
MN866227.1/Karnataka-State-India-Asia|2019
MN866240.1/Karnataka-State-India-Asia|2019
MN866227.1/Karnataka-State-India-Asia|2019
MN866241.1/Karnataka-State-India-Asia|2019
MN866227.1/Karnataka-State-India-Asia|2019
MN866228.1/Karnataka-State-India-Asia|2019
MN866227.1/Karnataka-State-India-Asia|2019
MN866243.1/Karnataka-State-India-Asia|2019
MN866227.1/Karnataka-State-India-Asia|2019
MN866235.1/Karnataka-State-India-Asia|2019
MN866227.1/Karnataka-State-India-Asia|2019
MN866221.1/Karnataka-State-India-Asia|2019
MN866224.1/Karnataka-State-India-Asia|2019
MN866238.1/Karnataka-State-India-Asia|2019
MN866233.1/Karnataka-State-India-Asia|2019
MN866220.1/Karnataka-State-India-Asia|2019
MN866242.1/Karnataka-State-India-Asia|2019
MN053754.1/Isla-Guadeloupe-America|2017
MN053757.1/Isla-Guadeloupe-America|2017
MN053760.1/Isla-Guadeloupe-America|2017
MN053763.1/Isla-Guadeloupe-America|2017
MN053766.1/Isla-Guadeloupe-America|2017
MN053778.1/Isla-Guadeloupe-America|2017
MN053781.1/Isla-Guadeloupe-America|2017
MN053745.1/Isla-Guadeloupe-America|2017
MN053772.1/Isla-Guadeloupe-America|2017
MN053775.1/Isla-Guadeloupe-America|2017
MN053751.1/Isla-Guadeloupe-America|2017
MN053769.1/Isla-Guadeloupe-America|2017
MN053757.1/Isla-Guadeloupe-America|2017
MN053748.1/Isla-Guadeloupe-America|2017
MN692603.1/Sao-paulo-America|2016
KM001085.1/Thailand-Asia|2008
LC498491.1/Accra-Ghana-Africa|2016
MF614132.1/Zhanjiang-China-Asia|2016
OQ305311.1/Ukunda-Kenya-Africa|2017
OR270144.1/Kilifi-Kenya-Africa|2021
OR270147.1/Kwale-Kenya-Africa|2021
MT361069.1/Kenya-Africa|2018
OQ305312.1/Ukunda-Kenya-Africa|2017
OQ305313.1/Ukunda-Kenya-Africa|2017
P- Val
8.00
4.00
0.00
BP Num
5.00
2,50
0.00
# Hits
5.00
2,50
0.00
1
1449
2898
4347
5796

**Fig. S2.** The analysis of *Phasivirus phasiense* using 60 genomic sequences identified specific regions with evidence of recombination events, enabling the detection of both parental and recombinant lineages [6]. Statistical significance values (*p*-values) were assigned to each event, supporting the robustness of the findings. The figure is structured into two panels: Subfigure A displays six distinct recombination events via breakpoint (BP) analysis, all localized within Asian countries (e.g., intra-country recombination between strains from Japan–Japan, China–China, and Thailand–Thailand). Subfigure B details the statistical validation, including BP numbering (BP Num: 6), hits (significant intra-regional signals), and corresponding *p*-values. Unlike the intercontinental patterns observed in other studies, these results emphasize localized genetic exchange, suggesting that recombination in *Phasivirus phasiense* predominantly drives diversification within geographically confined populations.
