# Supplementary material for: The genomic diversity of arthropod-specific viruses reinforces the continental distribution pattern of Aedes aegypti
Source: Parasit Vectors. 2025 Nov 18;18:468. doi: 10.1186/s13071-025-07120-3 (PMC12625191; doi:10.1186/s13071-025-07120-3)
Supplement: Supplementary file 2 — Additional file 2. Table S1. Geographic distribution and genetic data of insect-specific viruses (ISVs) in Ae. aegypti. [file 13071_2025_7120_MOESM2_ESM.pdf]

Supplementary Table 1. *Geographic Distribution and Genetic Data of Insect-Specific Viruses (ISVs) Analyzed in Aedes aegypti.*

This table includes the number of sequences analyzed for Phasivirus phasiense (PCLV), Aedes anphevirus (AeAV), and Cell-fusing agent virus (CFAV), along with their geographic origin and key genetic parameters. The "Host Provenance" column specifies the origin and rearing history of the mosquito from which each viral sequence was obtained. The categories are defined as follows:

- Wild: Mosquitoes collected as adults directly from their natural environment.
- Established colonies: Mosquitoes sourced from laboratory colonies that have been maintained for multiple generations.
- First-generation laboratory colonies: First-generation (F1) offspring reared in the laboratory from wild-caught parent mosquitoes.
- Laboratory strains of less than 2 generations: First or second-generation (F1-F2) offspring reared in the laboratory from wild-caught ancestors.
- Wild larvae, adults emerged in laboratory: Mosquitoes that were collected as larvae or pupae from field sites and subsequently reared to the adult stage under laboratory conditions before being processed.

#	Virus	Host	Continent	Country	City	ID_NCBI	Coverage %	Technology	Date	Assembly	Host Provenance
1	*Phasivirus phasiense*-L	*Aedes aegypti*	America	Brazil	Rio	KR003786.1	100	Illumina	2012	Velvet v 1.1.07	First-generation laboratory colonies
2	*Phasivirus phasiense*-L	*Aedes aegypti*	America	Brazil	Rio	NC_038262.1	100	Illumina	2012	Velvet v 1.1.07	First-generation laboratory colonies
3	*Phasivirus phasiense*-L	*Aedes aegypti*	America	Brazil	Sao Paulo	MN692603.1	99	Illumina	2016	SPAdes v3.13.1	Wild
4	*Phasivirus phasiense*-L	*Aedes aegypti*	America	Guadeloupe	Isla	MN053751.1	99	Illumina	2017	metaSPAdes v. 3.7	Wild
5	*Phasivirus phasiense*-L	*Aedes aegypti*	America	Guadeloupe	Isla	MN053745.1	99	Illumina	2017	metaSPAdes v. 3.7	Wild
6	*Phasivirus phasiense*-L	*Aedes aegypti*	America	Guadeloupe	Isla	MN053748.1	98	Illumina	2017	metaSPAdes v. 3.7	Wild
7	*Phasivirus phasiense*-L	*Aedes aegypti*	America	Guadeloupe	Isla	MN053781.1	98	Illumina	2016	metaSPAdes v. 3.7	Wild
8	*Phasivirus phasiense*-L	*Aedes aegypti*	America	Guadeloupe	Isla	MN053769.1	98	Illumina	2017	metaSPAdes v. 3.7	Wild
9	*Phasivirus phasiense*-L	*Aedes aegypti*	America	Guadeloupe	Isla	MN053757.1	98	Illumina	2017	metaSPAdes v. 3.7	Wild
10	*Phasivirus phasiense*-L	*Aedes aegypti*	America	Guadeloupe	Isla	MN053778.1	98	Illumina	2016	metaSPAdes v. 3.7	Wild
11	*Phasivirus phasiense*-L	*Aedes aegypti*	America	Guadeloupe	Isla	MN053754.1	98	Illumina	2017	metaSPAdes v. 3.7	Wild
12	*Phasivirus phasiense*-L	*Aedes aegypti*	America	Guadeloupe	Isla	MN053760.1	98	Illumina	2017	metaSPAdes v. 3.7	Wild
13	*Phasivirus phasiense*-L	*Aedes aegypti*	America	Guadeloupe	Isla	MN053772.1	96	Illumina	2017	metaSPAdes v. 3.7	Wild

14	*Phasivirus phasiense*-L	*Aedes aegypti*	America	Guadeloupe	Isla	MN053763.1	96	Illumina	2017	metaSPAdes v. 3.7	Wild
15	*Phasivirus phasiense*-L	*Aedes aegypti*	America	Guadeloupe	Isla	MN053766.1	96	Illumina	2017	metaSPAdes v. 3.7	Wild
16	*Phasivirus phasiense*-L	*Aedes aegypti*	Africa	Kenya	Kisumu	MT361069.1	99	Illumina	2018	BWA v.0.7.17	Wild
17	*Phasivirus phasiense*-L	*Aedes aegypti*	Asia	India	Karnataka State	MN866241.1	99	Illumina	2019	CLC Genomic Workbench v.11.1	Wild
18	*Phasivirus phasiense*-L	*Aedes aegypti*	Asia	India	Karnataka State	MN866238.1	99	Illumina	2019	CLC Genomic Workbench v.11.1	Wild
19	*Phasivirus phasiense*-L	*Aedes aegypti*	America	Guadeloupe	Isla	MN053775.1	93	Illumina	2017	metaSPAdes v. 3.7	Wild
20	*Phasivirus phasiense*-L	*Aedes aegypti*	Asia	India	Karnataka State	MN866231.1	99	Illumina	2019	CLC Genomic Workbench v.11.1	Wild
21	*Phasivirus phasiense*-L	*Aedes aegypti*	Asia	India	Karnataka State	MN866225.1	99	Illumina	2019	CLC Genomic Workbench v.11.1	Wild
22	*Phasivirus phasiense*-L	*Aedes aegypti*	Asia	India	Karnataka State	MN866226.1	99	Illumina	2019	CLC Genomic Workbench v.11.1	Wild
23	*Phasivirus phasiense*-L	*Aedes aegypti*	Asia	India	Karnataka State	MN866234.1	99	Illumina	2019	CLC Genomic Workbench v.11.1	Wild
24	*Phasivirus phasiense*-L	*Aedes aegypti*	Asia	China	Zhanjiang	MF614132.1	99	BGISEQ-500	2016	IDBA-UD v 1.1.1	Wild
25	*Phasivirus phasiense*-L	*Aedes aegypti*	Asia	India	Karnataka State	MN866236.1	99	Illumina	2019	CLC Genomic Workbench v.11.1	Wild
26	*Phasivirus phasiense*-L	*Aedes aegypti*	Africa	Kenya	Kwale	OR270147.1	97	Illumina	2021	SPAdes v. 3.15.5 and iVar v. 1.4.2	Wild
27	*Phasivirus phasiense*-L	*Aedes aegypti*	Asia	India	Karnataka State	MN866230.1	99	Illumina	2019	CLC Genomic Workbench v.11.0	Wild
28	*Phasivirus phasiense*-L	*Aedes aegypti*	Asia	India	Karnataka State	MN866222.1	99	Illumina	2019	CLC Genomic Workbench v.11.1	Wild
29	*Phasivirus phasiense*-L	*Aedes aegypti*	Asia	India	Karnataka State	MN866243.1	99	Illumina	2019	CLC Genomic Workbench v.11.0	Wild
30	*Phasivirus phasiense*-L	*Aedes aegypti*	Asia	India	Karnataka State	MN866239.1	99	Illumina	2019	CLC Genomic Workbench v.11.0	Wild
31	*Phasivirus phasiense*-L	*Aedes aegypti*	Africa	Kenya	Kilifi	OR270144.1	98	Illumina	2021	SPAdes v. 3.15.5 and iVar v. 1.4.2	Wild
32	*Phasivirus phasiense*-L	*Aedes aegypti*	Asia	India	Karnataka State	MN866223.1	99	Illumina	2019	CLC Genomic Workbench v.11.0	Wild
33	*Phasivirus phasiense*-L	*Aedes aegypti*	Asia	Thailand	X	KM001085.1	99	Illumina	2008	Trinity v. 2012-3-16	Wild
34	*Phasivirus phasiense*-L	*Aedes aegypti*	Asia	India	Karnataka State	MN866240.1	99	Illumina	2019	CLC Genomic Workbench v.11.0	Wild
35	*Phasivirus phasiense*-L	*Aedes aegypti*	Asia	India	Karnataka State	MN866235.1	99	Illumina	2019	CLC Genomic Workbench v.11.0	Wild
36	*Phasivirus phasiense*-L	*Aedes aegypti*	Asia	India	Karnataka State	MN866232.1	99	Illumina	2019	CLC Genomic Workbench v.11.0	Wild
37	*Phasivirus phasiense*-L	*Aedes aegypti*	Asia	India	Karnataka State	MN866229.1	99	Illumina	2019	CLC Genomic Workbench v.11.0	Wild
38	*Phasivirus phasiense*-L	*Aedes aegypti*	Asia	India	Karnataka State	MN866224.1	99	Illumina	2019	CLC Genomic Workbench v.11.0	Wild
39	*Phasivirus phasiense*-L	*Aedes aegypti*	Asia	India	Karnataka State	MN866237.1	99	Illumina	2019	CLC Genomic Workbench v.11.0	Wild
40	*Phasivirus phasiense*-L	*Aedes aegypti*	Asia	India	Karnataka State	MN866228.1	99	Illumina	2019	CLC Genomic Workbench v.11.0	Wild

41	*Phasivirus phasiense*-L	*Aedes aegypti*	Asia	India	Karnataka State	MN866221.1	99	Illumina	2019	CLC Genomic Workbench v.11.0	Wild
42	*Phasivirus phasiense*-L	*Aedes aegypti*	Asia	India	Karnataka State	MN866233.1	99	Illumina	2019	CLC Genomic Workbench v.11.0	Wild
43	*Phasivirus phasiense*-L	*Aedes aegypti*	Asia	India	Karnataka State	MN866220.1	99	Illumina	2019	CLC Genomic Workbench v.11.0	Wild
44	*Phasivirus phasiense*-L	*Aedes aegypti*	Asia	India	Karnataka State	MN866242.1	99	Illumina	2019	CLC Genomic Workbench v.11.0	Wild
45	*Phasivirus phasiense*-L	*Aedes aegypti*	Asia	India	Karnataka State	MN866227.1	81	Illumina	2019	CLC Genomic Workbench v.11.0	Wild
46	*Phasivirus phasiense*-L	*Aedes aegypti*	Africa	Ghana	Accra	LC498491.1	97	Illumina	2016	CLC Genomic Workbench v.11.0	Wild larvae, adults emerged in laboratory
47	*Phasivirus phasiense*-L	*Aedes aegypti*	Africa	Kenya	Kisauni	OQ305311.1	78	Illumina	2017	SPAdes v. 3.15.5 y Geneious v. 2023.0.1	Wild
48	*Phasivirus phasiense*-L	*Aedes aegypti*	Africa	Kenya	Ukunda	OQ305313.1	91	Illumina	2017	SPAdes v. 3.15.5 y Geneious v. 2023.0.1	Wild
49	*Phasivirus phasiense*-L	*Aedes aegypti*	Africa	Kenya	Ukunda	OQ305312.1	93	Illumina	2017	SPAdes v. 3.15.5 y Geneious v. 2023.0.1	Wild
50	*Phasivirus phasiense*-L	*Aedes aegypti*	America	Colombia	Ibague	Comuna06	99	Illumina	2021	Trinity v3.13.1	Wild
51	*Phasivirus phasiense*-L	*Aedes aegypti*	America	Colombia	Ibague	Comuna07	99	Illumina	2021	Trinity v3.13.1	Wild
52	*Phasivirus phasiense*-L	*Aedes aegypti*	America	Colombia	Ibague	Comuna08	99	Illumina	2021	Trinity v3.13.1	Wild
53	*Phasivirus phasiense*-L	*Aedes aegypti*	America	Colombia	Ibague	Comuna13	99	Illumina	2021	Trinity v3.13.1	Wild
1	*Aedes anphevirus*	*Aedes aegypti*	America	Guadeloupe	x	MN053738.1	99	Illumina	2017	metaSPAdes v. 3.7	Wild
2	*Aedes anphevirus*	*Aedes aegypti*	America	Guadeloupe	x	MN053739.1	98	Illumina	2016	metaSPAdes v. 3.7	Wild
3	*Aedes anphevirus*	*Aedes aegypti*	America	Guadeloupe	x	MN053740.1	99	Illumina	2017	metaSPAdes v. 3.7	Wild
4	*Aedes anphevirus*	*Aedes aegypti*	America	Guadeloupe	x	MN053737.1	99	Illumina	2017	metaSPAdes v. 3.7	Wild
5	*Aedes anphevirus*	*Aedes aegypti*	America	United States	Miami	MH430659.1	99	Illumina	2013	CLC Genomics Workbench v. 10.1.1	Wild
6	*Aedes anphevirus*	*Aedes aegypti*	America	Mexico	Chetumal	MH430658.1	99	Illumina	2013	CLC Genomics Workbench v. 10.1.1	Established colonies
7	*Aedes anphevirus*	*Aedes aegypti*	America	United States	California	MW435012.1	99	Illumina	2017	IDSeq pipeline v. 3.2	Wild
8	*Aedes anphevirus*	*Aedes aegypti*	America	Colombia	Cali	MH430650.1	98	Illumina	2013	CLC Genomics Workbench v. 10.1.1	Established colonies
9	*Aedes anphevirus*	*Aedes aegypti*	America	United States	California	MW435013.1	96	Illumina	2018	IDSeq pipeline v. 3.2	Wild
10	*Aedes anphevirus*	*Aedes aegypti*	Africa	Gabon	Bakoumba	MH430665.1	98	Illumina	2018	CLC Genomics Workbench v. 10.1.1	Laboratory strains of less than 2 generations
11	*Aedes anphevirus*	*Aedes aegypti*	Africa	Kenya	Rabai	MH430657.1	98	Illumina	2018	CLC Genomics Workbench v. 10.1.1	Laboratory strains of less than 2 generations

12	*Aedes anphevirus*	*Aedes aegypti*	Africa	Kenya	Rabai	MH430656.1	98	Illumina	2018	CLC Genomics Workbench v. 10.1.1	Laboratory strains of less than 2 generations
13	*Aedes anphevirus*	*Aedes aegypti*	Asia	Malaysia	Jinjang	MH430652.1	96	Illumina	x	CLC Genomics Workbench v. 10.1.1	Established colonies
14	*Aedes anphevirus*	*Aedes aegypti*	Asia	Thailand	Nakhon Nayok	MH430653.1	98	Illumina	x	CLC Genomics Workbench v. 10.1.1	Wild
15	*Aedes anphevirus*	*Aedes aegypti*	Asia	Thailand	Bangkok	MH430666.1	98	Illumina	x	CLC Genomics Workbench v. 10.1.1	Wild
16	*Aedes anphevirus*	*Aedes aegypti*	Asia	Thailand	Rayong	MH430655.1	98	Illumina	x	CLC Genomics Workbench v. 10.1.1	Laboratory strains of less than 2 generations
17	*Aedes anphevirus*	*Aedes aegypti*	Asia	Thailand	Chaiyaphum	MH430651.1	98	Illumina	x	CLC Genomics Workbench v. 10.1.1	Established colonies
18	*Aedes anphevirus*	*Aedes aegypti*	Oceania	French Polynesia	Bora Bora	MH430649.1	96	Illumina	x	CLC Genomics Workbench v. 10.1.1	Established colonies
19	*Aedes anphevirus*	*Aedes aegypti*	America	Colombia	Ibagué	Comuna06	96	Illumina	2021	Trinity v3.13.1	Wild
20	*Aedes anphevirus*	*Aedes aegypti*	America	Colombia	Ibagué	Comuna07	96	Illumina	2021	Trinity v3.13.1	Wild
21	*Aedes anphevirus*	*Aedes aegypti*	America	Colombia	Ibagué	Comuna08	96	Illumina	2021	Trinity v3.13.1	Wild
22	*Aedes anphevirus*	*Aedes aegypti*	America	Colombia	Ibagué	Comuna13	96	Illumina	2021	Trinity v3.13.1	Wild
1	*Cell fusing agent virus*	*Aedes sp*	America	Brazil	Macapá	MZ972993.1	99	Illumina	2017	Geneious v. R10	Wild
2	*Cell fusing agent virus*	*Aedes sp*	America	Brazil	Macapá	MZ972992.1	99	Illumina	2017	Geneious v. R10	Wild
3	*Cell fusing agent virus*	*Aedes aegypti*	America	United States	Florida	LR694072.1	99	x	2016	x	Wild
4	*Cell fusing agent virus*	*Aedes aegypti*	Africa	Uganda	Wakiso	LR694076.1	100	x	2016	x	First-generation laboratory colonies
5	*Cell fusing agent virus*	*Aedes aegypti*	Africa	Ghana	Accra	LC496857.1	99	x	2016	x	Wild larvae, adults emerged in laboratory
6	*Cell fusing agent virus*	*Aedes aegypti*	America	United States	Florida	LR694073.1	99	x	2016	x	Wild
7	*Cell fusing agent virus*	*Aedes sp*	America	Brazil	Macapá	MZ972994.1	99	Illumina	2017	Geneious v. R10	Wild
8	*Cell fusing agent virus*	*Aedes aegypti*	Asia	Cambodia	Kampong Cham	LR694077.1	100	x	2015	x	First-generation laboratory colonies
9	*Cell fusing agent virus*	*Aedes aegypti*	Asia	Cambodia	Kampong Cham	LR694079.1	99	x	2013	x	Wild
10	*Cell fusing agent virus*	*Aedes aegypti*	Asia	Cambodia	Kampong Cham	LR694080.1	100	x	2013	x	First-generation laboratory colonies
11	*Cell fusing agent virus*	*Aedes aegypti*	Asia	Cambodia	Phnom Penh	LR694078.1	100	x	2015	x	First-generation laboratory colonies
12	*Cell fusing agent virus*	*Aedes aegypti*	America	Guadeloupe	Les Abymes	LR694081.1	97	x	2016	x	Wild

13	*Cell fusing agent virus*	*Aedes aegypti*	Asia	Cambodia	x	OR479690.1	99	Illumina	2021	Megahit V v. 2022	Wild
14	*Cell fusing agent virus*	*Aedes aegypti*	America	Puerto Rico	Rio Piedras	GQ165810.1	91	x	2002	x	Wild
15	*Cell fusing agent virus*	*Aedes aegypti*	America	Mexico	x	KJ476731.1	89	Illumina	2011	ABySS v. 1	Wild
16	*Cell fusing agent virus*	*Aedes aegypti*	Asia	Cambodia	x	OR479689.1	93	Illumina	2021	Megahit V v. 2022	Wild
17	*Cell fusing agent virus*	*Aedes aegypti*	Asia	Cambodia	x	OR479687.1	93	Illumina	2021	Megahit V v. 2022	Wild
18	*Cell fusing agent virus*	*Aedes aegypti*	America	Colombia	Ibagué	Comuna06	98	Illumina	2021	Trinity v3.13.1	Wild
19	*Cell fusing agent virus*	*Aedes aegypti*	America	Colombia	Ibagué	Comuna07	98	Illumina	2021	Trinity v3.13.1	Wild
20	*Cell fusing agent virus*	*Aedes aegypti*	America	Colombia	Ibagué	Comuna08	98	Illumina	2021	Trinity v3.13.1	Wild
21	*Cell fusing agent virus*	*Aedes aegypti*	America	Colombia	Ibagué	Comuna09	98	Illumina	2021	Trinity v3.13.1	Wild
