# Supplementary material for: The genomic diversity of arthropod-specific viruses reinforces the continental distribution pattern of Aedes aegypti
Source: Parasit Vectors. 2025 Nov 18;18:468. doi: 10.1186/s13071-025-07120-3 (PMC12625191; doi:10.1186/s13071-025-07120-3)
Supplement: Supplementary file 3 — Additional file 3. Table S2. Genetic diversity parameters of ISVs by continent. [file 13071_2025_7120_MOESM3_ESM.pdf]

Supplementary Table 2. Genetic diversity of evaluated ISVs. The table summarizes various genetic and diversity parameters for three viral species for continent: Aedes anphevirus (AeAV), Cell-fusing agent virus (CFAV), and Phasivirus phasiense (PCLV). SD = Standard deviation, SD_NR= Standard deviation of theta (no recombination).

Americas

Parameter	AeAV	CFAV	PCLV
Number of sequences	13	12	20
Observation window (bp)	12271	4951	5796
Polymorphic sites (S)	448	639	361
Nucleotide diversity (π ± SD)	0.01197 ± 0.0006	0.0299 ± 0.0050	0.01318 ± 0.0006
Number of haplotypes	12	12	18
Haplotype diversity	0.987	1	0.989
Theta-W (per site ± SD_NR)	0.0117 ± 0.0000506	0.03216 ± 0.02079	0.01756 ±0.00092
Theta-W (per sequence ± SD_NR)	144.367 ± 7,13	201.249 ± 73.24	101.755 ± 38.56
Sites with gaps/missing data	28	3	2

Asia

Parameter	AeAV	CFAV	PCLV
Number of sequences	5	7	26
Observation window (bp)	12291	4951	5796
Polymorphic sites (S)	388	231	526
Nucleotide diversity (π ± SD)	0.01329 ± 0.126	0.01684 ± 0.00256	0.01767 ± 0.000002
Number of haplotypes	5	7	25

Haplotype diversity	1	1	0.997
Theta-W (per site ± SD_NR)	0.015 ± 0.0000572	0.01905 ± 0.0000735	0.02379 ± 0.0000572
Theta-W (per sequence ± SD_NR)	189.240 ± 10.61	94.286 ± 42.43	137.842 ± 43.81
Sites with gaps/missing data	8	1	1

Africa

Parameter	AeAV	CFAV	PCLV
Number of sequences	3	2	7
Observation window (bp)	12292	4951	5796
Polymorphic sites (S)	201	127	196
Nucleotide diversity (π ± SD)	0.0109 ± 0.0000101	0.02566 ± 0.01283	0.01078 ± 0.00397
Number of haplotypes	3	2	7
Haplotype diversity	1	1	1
Theta-W (per site ± SD_NR)	0.0109 ± 0.0000428	0.02566 ±0.0003	0.01393 ±0.00628
Theta-W (per sequence ± SD_NR)	134 ± 80.44	127 ± 90.16	80.00 ± 36.62
Sites with gaps/missing data	7	1	55
